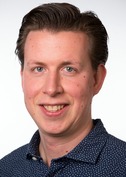# Electrolyte Effects on the Electrochemical Reduction of CO_2_

**DOI:** 10.1002/cphc.201900680

**Published:** 2019-11-07

**Authors:** Marilia Moura de Salles Pupo, Ruud Kortlever

**Affiliations:** ^1^ Department of Process & Energy Faculty of Mechanical, Maritime & Materials Engineering Delft University of Technology Leeghwaterstraat 39 2628 CB Delft, The Netherlands

**Keywords:** CO_2_ reduction, CO_2_ utilization, electrocatalysis, electrolyte effects, electroreduction

## Abstract

The electrochemical reduction of CO_2_ to fuels or commodity chemicals is a reaction of high interest for closing the anthropogenic carbon cycle. The role of the electrolyte is of particular interest, as the interplay between the electrocatalytic surface and the electrolyte plays an important role in determining the outcome of the CO_2_ reduction reaction. Therefore, insights on electrolyte effects on the electrochemical reduction of CO_2_ are pivotal in designing electrochemical devices that are able to efficiently and selectively convert CO_2_ into valuable products. Here, we provide an overview of recently obtained insights on electrolyte effects and we discuss how these insights can be used as design parameters for the construction of new electrocatalytic systems.

## Graphical abstract

**The electrochemical reduction** of CO_2_ to fuels and chemicals can potentially close the carbon cycle. Here, the authors discuss the importance of the electrolyte in determining the outcome of the reaction. They highlight recently obtained insights on electrolyte effects and discuss how the electrolyte choice can be used as a design parameter for more efficient electrocatalytic systems.

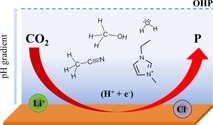

## 1 Introduction

The electrochemical reduction of CO_2_ to fuels or commodity chemicals, driven by renewable energy, provides a unique opportunity to both utilize CO_2_ and store renewable energy in chemical bonds.^1^, ^2^, ^3^ Although the electrochemical reduction of CO_2_ to valuable products is promising, since it can be performed at room temperature and atmospheric pressure using earth abundant electrocatalytic materials, major issues need to be resolved before the process becomes feasible.^4^ Many of these issues are related to the high overpotentials needed for product formation, poor product selectivity, low conversion rates, and poor stability of the catalytic system. Until now, most research has focused on the development of novel electrocatalysts for CO_2_ reduction, since the observed overpotentials and poor product selectivity stem from the suboptimal binding of key intermediates on the catalytic surface.^5^ Obviously, these novel electrocatalysts will only perform optimally under optimized process conditions. One of the important factors determining the outcome of CO_2_ reduction is the interplay between the electrocatalytic surface and the electrolyte. Therefore, in‐depth understanding of the electrolyte effects on CO_2_ electrocatalysis is crucial in designing efficient systems for the reduction of CO_2_ to desired products. This minireview aims to provide an overview of recently obtained insights regarding electrolyte effects on the electrochemical CO_2_ reduction reaction (CO_2_RR) and to give an outlook on how these insights can be used as design parameters to lower overpotentials for CO_2_ reduction and boost product selectivity.

### 1.1 Electrochemical CO_2_ Reduction

Ever since the landmark discovery by Hori in 1985, who found that copper electrodes are able to electrochemically reduce CO_2_ to hydrocarbons, ample research has been dedicated to understanding and improving this reaction.^6^, ^7^ Although copper is able to produce hydrocarbons from CO_2_, the product distribution on other metals is mostly restricted to CO and formate, both 2e^−^ transfer products. In general, CO production is dominant on transition metals such as Au, Ag and Zn, while formate production is dominant on *p*‐block metals such as Sn, Pb and In.^8^, ^9^, ^10^ In most cases, significant overpotentials are required to drive the electrochemical reduction of CO_2_ and obtain products. For CO_2_ reduction on copper surfaces the production of 16 different products has been reported, with low product selectivity.^11^ This observation exemplifies the challenges and opportunities of electrochemical CO_2_ reduction. In addition to the high overpotentials and poor product selectivity, the overall efficiency of electrochemical CO_2_ reduction suffers from competition with the hydrogen evolution reaction (HER), occurring in the same potential window.

To overcome these challenges, much effort has been invested in designing and optimizing electrocatalysts for CO_2_ reduction. Examples of these efforts are studies working with size‐selected and shape‐controlled nanoparticles,^12^, ^13^, ^14^ nano‐structured and oxide‐derived surfaces^15^, ^16^, ^17^ and bimetallic electrocatalysts.^18^, ^19^, ^20^, ^21^ The design of new electrocatalysts has been aided by increased mechanistic understanding on how electrochemical CO_2_ reduction takes place on heterogeneous electrocatalytic surfaces, mostly obtained by spectro‐electrochemical and computational studies.^22^, ^23^, ^24^, ^25^

The next step toward implementation of electrochemical CO_2_ reduction is the integration of electrocatalysts and other materials into electrochemical systems and reactors.^26^, ^27^ These systems operate at high current densities and will preferably have both high product selectivities and high energy efficiencies. To achieve these goals, optimal process conditions in the electrochemical reactor are key. Therefore, the selection of the right electrolyte will be paramount for the successful operation of an electrochemical system that is able to selectively and efficiently convert CO_2_ into products.

## 2 CO_2_ Reduction in Aqueous Electrolytes

Most studies on the electrocatalytic reduction of CO_2_ employ aqueous electrolytes.^28^ The overall solubility of CO_2_ in water is however low, approximately 34 mM at standard conditions. The addition of salts to form the aqueous electrolyte can induce a salting out effect, further lowering CO_2_ solubility in the electrolyte.^29^ Therefore, if CO_2_ is solely present as dissolved CO_2_ in an electrolyte, this limited solubility will induce mass transfer limitations during operation at higher current densities. Gas diffusion‐based setups and membrane electrode assembly reactors with a gas phase can provide solutions to this problem, as these setups create a triple‐phase boundary where gaseous CO_2_ is in contact with an electrolyte close to the electrocatalytic surface. This allows for fast diffusion of CO_2_ towards the electrocatalytic surface. Thereby, these system are able to sustain significantly higher current densities than systems where CO_2_ is dissolved in the bulk electrolyte, e. g. a traditional H‐cell.^26^

A general concern is the purity of the electrolyte, as trace metal impurities can deposit on the electrocatalytic surface altering the efficiency and selectivity of the process over time. To mitigate this effect, the electrolyte can be purified by using a pre‐electrolysis method.^30^ By using this method, any residual metal ions in the electrolyte are deposited on a sacrificial electrode before the electrolyte is used for CO_2_ reduction experiments. An alternative method, recently demonstrated by Wuttig et al., uses metal ion complexation to clean the electrolyte and enhance long‐term stability.^31^

### 2.1 Effect of the Electrolyte pH

The local pH at the electrode interface is an important parameter controlling the electrocatalytic selectivity.^32^ Due to the formation of OH^−^ by both CO_2_ reduction and the competing hydrogen evolution reaction, the local pH near the surface of the electrode will differ from the pH of the bulk electrolyte. This effect can be counteracted to some extent by using buffering electrolytes. In fact, CO_2_ itself forms a bicarbonate buffer in aqueous electrolytes:(1)
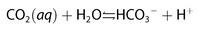



or in basic electrolytes with a pH higher than 7;(2)
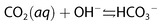



Moreover, the bicarbonate buffer can neutralize OH^−^ generated at the electrode surface:(3)
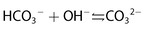



The local pH at the electrode surface is thus dependent on the current density, and more specifically the partial current density toward the formation of different products and H_2_ as side‐product, the buffering strength of the electrolyte and the mass‐transport of OH^−^, CO_2_, HCO_3_^−^ and CO_3_^2−^. This means that even for a flat metal foil electrode in a buffered 0.1 M KHCO_3_ electrolyte, the local pH at the surface is significantly higher than the pH of the bulk electrolyte.^34^, ^35^

**Figure 1:**
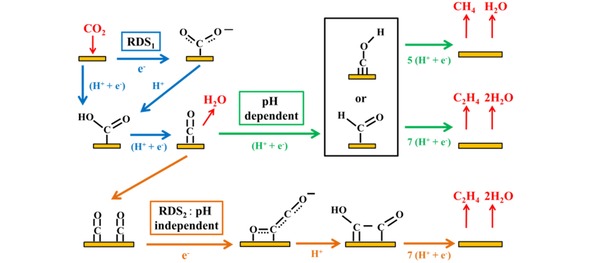
Possible reaction pathways for the production of methane and ethylene from CO_2_ and CO on copper surfaces: In blue, reduction of CO_2_ to CO; in green, the pH dependent C_1_ pathway that forms methane and ethylene via dimerization of intermediates; in orange the pH independent C_2_ pathway that produces ethylene.^**[33]**^ Species in black represent adsorbates, while species in red represent reactants and products in solution. RDS indicates rate determining steps while (H^+^+e^−^) or H^+^ and e^−^ indicate steps with either concerted proton‐electron transfer or separated proton‐electron transfer.

The local pH will affect the competing hydrogen evolution reaction as this reaction is pH dependent. Moreover, work by Hori et al. showed that the local pH also plays an important role in the electrochemical reduction of CO_2_ to hydrocarbons on copper electrodes.^36^ While the formation of methane was found to be pH sensitive, the formation of ethylene was found to be pH insensitive, suggesting separate pathways for the production of these hydrocarbons. Later work by the Koper group indeed showed that there are two pathways for the production of hydrocarbons; a pH dependent C_1_ pathway, depicted as the green pathway in Figure 1, that primarily produces methane and forms ethylene by dimerization of intermediates and a pH independent C_2_ pathway, depicted as the orange pathway in Figure 1, that produces ethylene via the formation of a CO dimer intermediate.^22^, ^24^

**Figure 2:**
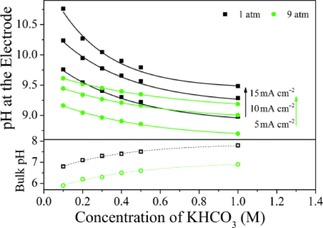
Estimated local pH at a rough copper surface at different current densities as a function of bicarbonate concentration in the electrolyte and CO_2_ pressure. Corresponding bulk pH of the electrolyte for different bicarbonate concentrations (independent of current density). Reproduced with permission from Kas et al.^37^

Knowing this, one can take advantage of the difference in pH dependence of these two pathways. Kas et al. analysed the influence of bicarbonate concentration, CO_2_ pressure and current density on the local pH at the electrode surface and the selectivity of methane and ethylene formation (see Figure 2).^37^ In doing so, they were able to tune the interfacial pH to reach optimal conditions for the selective production of ethylene, reaching Faradaic efficiencies (FE) of 44 % toward ethylene production and 2 % toward methane production in a 0.1 M KHCO_3_ electrolyte applying −1.1 V (vs Ag/AgCl) at 9 atm.^37^ Varela et al. found that electrolytes with a higher bicarbonate concentration allow for a stronger buffer effect, effectively lowering the local pH, and, while working under the same conditions as Kas et al., methane was observed as the main product.^37^, ^38^

To selectively produce ethylene and ethanol, one would like to make optimal use of the pH dependence and perform CO_2_ reduction in alkaline media. However, due to the equilibrium reactions of CO_2_ and OH^−^ to form bicarbonate and carbonate (equation 2 and 3) this is impossible. To overcome this limitation, Cook et al. used a gas diffusion electrode to separate the reactant CO_2_ from a stationary KOH electrolyte.^39^ At a current density of 400 mA/cm^2^ and a cathode potential of −1.98 V vs. RHE they observed a Faradaic efficiency of 9.1 % toward methane production and 69 % toward ethylene production. More recently, the Kenis group and the Sargent group have further explored these alkaline electrolyser systems by optimizing electrode materials and introducing electrolyte flow.^40^, ^41^, ^42^, ^43^, ^44^ Overall, high Faradaic efficiencies toward ethylene production on copper based electrodes, up to 70 %, and toward CO production on silver based electrodes, up to 84 %, can now be achieved at significantly lower cathode potentials. A downside of the alkaline electrolyser approach is loss of reactant CO_2_ by a side reaction with the alkaline electrolyte at the triple phase boundary forming (bi)carbonates. This results in acidification of the electrolyte and thus electrolyte degradation.

### 2.2 Cation Effects

**Figure 3:**
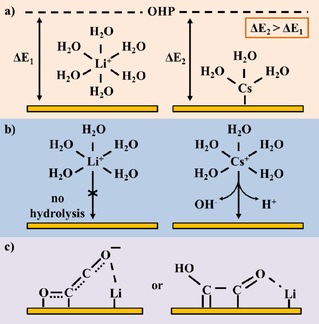
Possible explanations for the observed cation effect on CO_2_ electrocatalysis: a) Change in outer Helmholtz plane (OHP) potential induced by cation size and specific adsorption on the electrocatalytic surface; b) Hydrolysis of water molecules comprising the hydration shell of the cation in the vicinity of the electrocatalytic surface; c) Cation interaction with adsorbed intermediates on the electrocatalytic surface, where the cation acts as a catalytic promotor.

Early work by Murata and Hori showed that the selectivity of CO_2_ reduction on copper electrodes is highly dependent on the cations present in the electrolyte.^45^ They observed that with increasing Stokes radii of the hydrated cations, from Li^+^ to Cs^+^, the selectivity toward C_2_ products increases. This observation was rationalized by the change in the outer Helmholtz plane (OHP) potential induced by varying the cation size. Smaller cations, such as Li^+^, are strongly hydrated, preventing specific adsorption of the cation on the electrode surface. Larger cations will adsorb more easily on the electrode surface, leading to a change in OHP potential. Since protons are a charged species, the change in OHP potential will affect the proton concentration at the electrode interface, leading to the observed change in hydrocarbon selectivity (see Figure 3a). Moreover, Resasco et al. reported that the change in OHP potential could also stabilize surface‐bound intermediates, leading to differences in reaction rates for the formation of products.^46^ Recently, Ringe et al. have modelled the impact of cations on the interfacial electric field. They find that their model is able to correlate changes in the interfacial electric field to experimentally observed cation effects on CO_2_ reduction on gold and copper surfaces.^47^

Similar cation effects are reported on silver electrocatalysts, where a decrease in H_2_ formation is observed with increasing cation radii.^48^, ^49^ Singh et al. postulated that the effects on both silver and copper electrocatalyst are caused by cation hydrolysis near the electrode surface (see Figure 3b).^49^ They show that the dissociation of water molecules making up the hydration shell of a cation is dependent on the electrostatic interactions. Thereby, it is possible that protons are formed by water dissociation once the cation is in close proximity to the electrode surface. This mechanism is dependent on cation radius and will alter the local pH and the local CO_2_ concentration near the electrode surface, ultimately altering product selectivity. Ayemoba and Cuesta have further confirmed this effect by performing surface‐enhanced infrared spectroscopy (SEIRAS) experiments on a thin‐film gold electrode.^50^

Pérez‐Gallent et al. provide an alternative explanation to the cation‐induced change in hydrocarbon selectivity observed on copper electrodes (see Figure 3c).^51^ With smaller cations, such as Li^+^, Na^+^ and K^+^, present in the electrolyte a hydrogenated dimer intermediate (OCCOH) is observed using infrared spectroscopy during CO reduction experiments.^51^, ^52^ However, this hydrogenated dimer is not observed when larger cations, Rb^+^ and Cs^+^, are present in the electrolyte. The cation effect is therefore explained as a catalytic promotor effect, where the cations are able to specifically stabilize certain intermediates, thereby changing the free energy landscape of the reduction reaction. These experimental observations are supported by computational work by Akhade et al. who report a similar promotor effect with specifically adsorbed K^+^ co‐adsorbed on a copper surface.^53^ A similar promotor effect is reported for molecular catalysts adsorbed on a heterogeneous surface, where adsorbed K^+^ stabilizes a CO_2_ adduct on the metal center.^54^

### 2.3 Anion Effects

**Figure 4:**
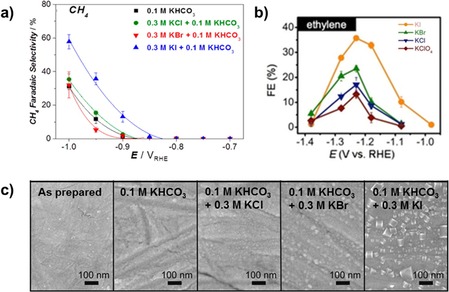
Anion effects on electrochemical CO_2_ reduction: a) Enhanced Faradaic efficiency towards methane production on copper electrodes in 0.1 M KHCO_3_ electrolytes with different halide anions present; b) Enhanced Faradaic efficiency towards ethylene on a Cu(111) electrode in pure halide electrolytes; c) Morphology changes of copper foil electrodes after CO_2_ reduction experiments in different (halide containing) electrolytes, most left image shows the copper surface, prepared via mechanical polishing, before electrolysis for comparison. Figure 4a) and c) are adapted and reprinted with permission from Varela et al, *ACS Catal.,* **2016**, *6*, 2136–2144.^57^ Copyright 2016 American Chemical Society. Figure 4b) is adapted and reprinted with permission from Huang et al.^59^

Besides cations, anions have a significant effect on the outcome of electrocatalytic CO_2_ reduction as well.^55^ Most studies have focused on the influence of halide anions on CO_2_ reduction.^56^, ^57^, ^58^, ^59^ Varela et al. studied the effect of halide anions on the reduction of CO_2_ by adding halide salts to 0.1 M KHCO_3_ electrolytes.^57^ Their study shows that the addition of Cl^−^, Br^−^ and I^−^ has an effect on the activity and selectivity of CO_2_ reduction on copper, depending on the concentration and nature of the added halide. In the case of Cl^−^ and Br^−^ addition, an increase in CO selectivity is observed, while a decrease in CO selectivity and an enhancement of methane formation is observed when I^−^ is added to the electrolyte (see Figure 4a). Moreover, as shown in Figure 4c the presence of Br^−^ and I^−^ anions in the electrolyte induces morphology changes of the copper surface. The observed activity and selectivity changes are attributed to the specific adsorption of the anions on the catalytic surface, increasing the negative charge on the surface.^60^

In contrast to the work by Varela et al., an increase in C_2_ product selectivity is observed during experiments on copper in pure halide electrolytes, without HCO_3_^−^ present (see Figure 4b).^56^, ^59^ Huang et al. studied the reduction of CO_2_ on copper single‐crystal electrodes, Cu(100) and Cu(111), in halide containing electrolytes using in situ Raman spectroscopy and show that the presence of halide anions alters the coordination environment of surface‐bound CO.^59^ Overall, this effect is strongest with I^−^ present in the electrolyte, hence yielding the highest increase in selectivity toward C_2_ with I^−^.

The presence of perchlorate, sulfate, phosphate and borate did not significantly affect the production of CO, HCOO^−^, C_2_H_4_ and CH_3_CH_2_OH on copper electrodes.^58^ However, the production of H_2_ and CH_4_ was found to be very sensitive to the nature and concentration of these anions. In comparison to bicarbonate electrolytes, the selectivity towards H_2_ and CH_4_ increased when phosphate and borate anions were present, while it decreased when perchlorate and sulfate were present in the electrolyte. This observation was attributed to the ability of these buffering anions to serve as a proton donor. Therefore, using anions with a low buffering capacity will result in a suppression of H_2_ and CH_4_ production. This hypothesis is supported by recent work of Jackson et al., that shows that phosphate can outcompete water as a proton donor in the hydrogen evolution reaction on a gold electrode.^61^

## 3 CO_2_ Reduction in Non‐Aqueous Electrolytes

Attempts to improve the overall efficiency of electrochemical CO_2_ reduction have employed several non‐aqueous electrolytes.^62^, ^63^, ^64^ The main advantage of using non‐aqueous electrolytes such as acetonitrile, dimethyl formamide and methanol is that these electrolytes generally have a higher CO_2_ solubility than aqueous electrolytes.^62^, ^65^, ^66^, ^67^ Additionally, these solvents have lower proton concentrations, suppressing the unwanted generation of hydrogen as a side‐product during CO_2_ reduction. Moreover, alternative reaction pathways can occur in non‐aqueous electrolytes that favor the formation of specific products.^41^

When working with non‐aqueous electrolytes it is important to take into account that the carbon atoms incorporated in detected products can stem from either CO_2_ reduction or from the carbon atoms present in the electrolyte.^68^ Therefore, it is recommended that proper precautions are taken to verify that the products formed are in fact produced from CO_2_ fed into the electrolyte prior to the experiment.^69^ Isotopic labelling has proven to be an effective method to determine the origin of the carbon atoms found in assumed CO_2_ reduction products. Using this method, one can distinguish carbon atoms that originate from dissolved ^13^CO_2_ from carbon atoms that stem from ^12^C‐containing electrode materials or electrolytes.^70^ In fact, isotopic labelling has become the golden standard to determine the origin of the products formed and is recommended to be used, potentially in combination with FTIR, to determine the source of carbonaceous gaseous products. Additionally, in cases where CO_2_RR products present very low Faradaic efficiencies, isotopic labelling can be especially useful to identify possible cases of cross contamination in the experimental setup saving time and overall costs.^71^

When working with non‐aqueous electrolytes, interference of carbon atoms from different sources than CO_2_ becomes particularly relevant, as these electrolytes are capable of interacting with the CO_2_RR in different ways. For example, studies on the electrochemical production of dimethyl carbonate from CO_2_ dissolved in acetonitrile (99.85 % anhydrous from Sigma Aldrich), using Ag/Ag^+^ as a reference electrode and Cu, Pt and Pb as working electrodes and a Pt coil as counter electrode relied on isotopic labelling to determine the reaction pathway. In this study, it was shown that, contrary to what is generally reported, during the electrosynthesis of organic carbonates the reduced form of CO_2_ participating in the carboxylation step is CO and not the CO_2_^.−^ radical.^72^

### 3.1 Organic‐Solvent‐Based Electrolytes

In general, the electrochemical reduction of CO_2_ in aprotic electrolytes follows three different pathways: 1) The formation of oxalic acid via dimerization of two CO_2_^.−^ molecules; 2) a disproportionation reaction where CO_2_^.−^ and CO_2_ disproportionate to form CO and CO_3_^2−^ and 3) in the presence of small amounts of water, the formation of formic acid due to protonation of a CO_2_^.−^ radical followed by electron transfer.^73^, ^74^, ^75^ In protic electrolytes, hydrogen evolution can occur and the formation of hydrocarbon products on copper electrodes is observed. Thus, electrolyte choice actively influences both the product distribution and the product selectivity depending on the capability of the electrolyte to act as proton donor. Table 1 provides an overview of organic solvent‐based electrolytes and the main product obtained from the CO_2_RR using different electrode materials.

**Table 1:** CO_2_RR in non‐aqueous electrolytes, highlighting the electrode material, electrolyte and main products obtained from the electrochemical reduction of CO_2_.

Electrode	Electrolyte	Main product (FE%)	Ref
Cu foil	0.08 M NaOH in methanol (catholyte) 0.3 M KOH in methanol (anolyte)	Methane (80.6 %)	60
Cu foil	0.5 M CH_3_COOLi, LiBr, LiCl, LiClO_4_ in methanol (catholyte) 0.3 M KOH in methanol (anolyte)	Methane (71.8 %)	76
Cu wire	0.5 M LiClO_4_ in methanol	Methane (37.5 %) and CO (48.3 %)	77
Au foil	0.1 M KOH in methanol	CO (52.7 %) and Formic Acid (13.8 %)	78
Pt mesh	0.01 M benzalkonium chloride in methanol (catholyte) 0.1 M KHCO_3_ in water (anolyte)	Methane (3 %) and ethylene (0.3 %)	65
Co_3_O_4_ nanofibers	Acetonitrile and 1 % (w/v) H_2_O	CO (65 %) and Formate (27 %)	79
Ag foil	0.1 M DMF and 0.5 % (v/v) H_2_O	CO (95 %)	80

The solubility of CO_2_ in acetonitrile is around 270 mM, which is eight times higher than the CO_2_ solubility in aqueous solutions.^62^ In this polar, aprotic electrolyte the main product formed is CO.^79^

However, it is important to keep in mind that CO_2_ reduction in acetonitrile is extremely sensitive to the presence of water. Shifts in reaction pathways and products selectivities occur even in the presence of trace amounts of water (as low as 46 ppm). Aljabour et al. studied CO_2_ reduction on Co_3_O_4_ nanofibers in acetonitrile with small amounts of water (1 % w/v).^79^ In accordance with earlier discussed reaction pathways, they observe that in water containing electrolytes formate is produced, while in pure acetonitrile CO is the main product formed via the disproportionation of CO_2_^.−^ and CO_2_. Additionally, the presence of water has been shown to lead to the decomposition of acetonitrile into acetamide and to promote the formation of (bi)carbonates from CO_2_.^63^

CO_2_ solubility in methanol electrolytes at room temperature can be five times higher than the CO_2_ solubility in water.^81^ At lower temperatures CO_2_ solubility in methanol can be further increased, to up to fifteen times higher solubilities than in water. The electrochemical CO_2_ reduction in methanol tends to preferentially produce CO, methane and ethylene as demonstrated by studies carried out on Pt mesh electrodes.^67^ Additionally, since methanol is already used in the Rectisol CO_2_ absorption process, the combination with an electrochemical CO_2_ conversion method could combine CO_2_ capture and utilization.^76^

Comparative studies have shown that methanol‐based electrolytes are among the best non‐aqueous electrolytes to obtain hydrocarbons (e. g. methane and ethylene). Moreover, the addition of cations to the electrolyte has proven to be a simple and direct route of tailoring the products formed during CO_2_RR. For example, Kaneco et al., studied electrochemical CO_2_ reduction in methanol with 80 mM NaOH on Cu foils and found relatively high Faradaic efficiencies for the production of methane ranging from 47 % to 62 % when working at potentials from −2.0 to −3.0 V vs. Ag/AgCl.^62^

Unfortunately, although most organic‐solvent based electrolytes are capable of significantly suppressing HER, they generally present very low Faradaic efficiencies toward CO_2_ reduction products. This limits the perspectives of organic solvent‐based electrolytes for CO_2_ reduction in large‐scale applications.^82^

### 3.2 Ionic‐Liquid‐Based Electrolytes

As most ionic liquids have appreciable electric conductivities, it is possible to apply them as electrolyte without the addition of water.^83^, ^84^, ^85^ Some studies consider certain ionic liquids (e. g. [BMIm][PF_6_]) both as an electrolyte and a promotor for CO_2_ activation. By lowering the free energy for the formation of the CO_2_^.−^ intermediate, ionic liquids can reduce the initial barrier of CO_2_ reduction.^85^ Additionally, due to their ionic interchanging fields, ionic liquids are known for having a higher CO_2_ solubility. Next to that, some ionic liquids can form a CO_2_ adduct. The formation of these adducts also increases CO_2_ diffusion rates with respect to aqueous solutions.^86^ Moreover, the capability of ionic liquids to absorb CO_2_ physically and chemically can open a variety of options to efficiently convert CO_2_ into valuable chemical products.^85^ Using ionic liquids at temperatures below room temperature will increase CO_2_ solubility, but has also been reported to decrease mass transport as the viscosity of ionic liquids increases with decreasing temperatures.^87^

The wide variety of commercially available ionic liquids allows for tailoring of the electrolyte toward the most favorable conditions for electrochemical CO_2_ reduction. Zhou et al.^88^ carried out comparative tests, studying electrochemical CO_2_ reduction in a variety of ionic liquids with various concentrations of water (20, 40, 60 and 80 wt %) using Ag, Au, Cu and Pt as electrode materials. From all the ionic liquids tested, the most selective system for the electrochemical reduction of CO_2_ to CO was found to be an Ag electrode in a BMIm‐Cl electrolyte containing 20 wt % H_2_O. With an overall current density of 2.4 mA cm^−2^, a CO selectivity of >99 % was reached. This result is thought to be due to the strong hydrogen bonds formed between the hydrogen atoms in water and Cl^−^, thereby inhibiting the HER reaction.

**Figure 5:**
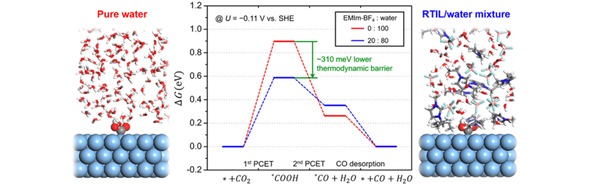
Calculated reaction free energy profiles during CO_2_‐to‐CO reduction in aqueous and 20 : 80 EMIm‐BF_4_/water mixed electrolytes at applied potential *U*=−0.11 V vs SHE. Reprinted with permission from Lim et al, *ACS Catal.,* **2018**, *8*, 2420–2427.^[90]^ Copyright 2018 American Chemical Society.

Electrochemical CO_2_ reduction on Ag electrodes in ionic liquids favors the formation of CO.^64^, ^89^ Liu et al. show that performing CO_2_ reduction on Ag_2_S nanowires in a [EMIm]^+^ based ionic liquid improves the partial current density for CO production by a 14‐fold in comparison to CO_2_ reduction on Ag_2_S nanowires in an aqueous KHCO_3_ electrolyte. This difference is explained by the formation of [EMIm‐CO_2_]^+^ complexes as intermediates.^89^ These [EMIm‐CO_2_]^+^ complexes adsorb on the electrocatalytic surface, thereby increasing the local CO_2_ concentration near the surface. Additionally, the complex can react with water and form [EMIm‐HCO_3_] or [EMIm‐CO_3_]^−^ species that can adsorb on the cathode surface and can potentially reduce energy barriers for the reduction of CO_2_.^87^ Additional studies carried out by Lim et al. have shown through systematic experiments that cations and anions in room temperature ionic liquids are capable of stabilizing surface bound intermediates, resulting in a suitable microenvironment that lowers energy barriers and improves CO_2_ reduction kinetics (see Figure 5).^90^

Michez et al. investigated the stability of ionic liquid electrolytes by studying the reductive decomposition of BMIm‐NTf_2_ at gold electrodes.^91^ Using NMR, they identify intermediaries formed by the decomposition of the ionic liquid. Also, they find that CO_2_ electroreduction takes place at potentials that are very close to the cathodic limit of the ionic liquid. By pre‐electrolyzing the ionic liquid before electrochemical CO_2_ reduction, changes in the CO_2_RR were found showing that the decomposition of the ionic liquid actively influences CO_2_ reduction. Although ionic liquids are generally considered to be highly stable, this study demonstrates that it is important to account for the cathodic limit of the ionic liquid, as electrolyte decomposition will affect the obtained results.

## 4 CO_2_ Reduction in Electrolyte Mixtures

Some studies have investigated CO_2_ reduction in electrolyte mixtures.^79^, ^88^, ^92^ Here, we have classified electrolyte mixtures as electrolytes containing two different components, where one of the components accounts for at least 5 % of the electrolyte. Electrolytes that contain a very low amount of water are quite commonly reported in literature^79^, ^80^ and were considered as non‐aqueous electrolytes (see Table 1). Although using a non‐aqueous electrolyte can lead to improvements in the solubility of CO_2_, the overall lack of a proton donor is considered a limiting factor for the CO_2_ reduction reaction. Therefore, studies using electrolyte mixtures generally report increased reaction rates compared to reactions using non‐mixed electrolytes.^62^, ^79^, ^93^ Table 2 presents a selection of CO_2_RR studies carried out using various mixed electrolytes and electrodes presenting their main products.

**Table 2:** Electrochemical CO_2_ reduction in mixed electrolytes, highlighting the electrode material, electrolyte composition and main products obtained.

Electrode	Electrolyte	Main product (FE%)	Ref
Pt, Au and Pb	Acetonitrile and H_2_O (3.94 mM–2356 mM)	Oxalic Acid (72 %), CO (18 %) and Formic Acid (10 %)	93
BDD films	1 M NH_3_ and 0.1 M NH_4_HCO_3_ in H_2_O	Methanol (24.3 %)	95
Ag, Au, Cu and Pt	ILs and H_2_O, 20–80 % w/v	CO (99 %)	88
Ag	EMIm and H_2_O 0–98 mol %	CO (99 %)	92
Cu dendrites	[EMIm]BF_4_ and H_2_O, 92–8 % v/v	HCOOH (87 %)	96
Hg	DMF and BuNClO_4_ 0.2 M	CO (67 %) and oxalate (25 %)	97

Tomita et al. studied electrochemical CO_2_ reduction in acetonitrile‐water mixtures using Pt electrodes.^93^ In aqueous systems, CO_2_ reduction on Pt electrodes is poisoned by the formation of a layer of tightly bound CO adsorbates. Additionally, Pt is a good electrocatalyst for HER and subsequently CO_2_ reduction experiments using Pt in aqueous media mainly yield hydrogen. However, working with acetonitrile‐water mixtures shows that the product distribution is very much dependent on the electrolyte composition. While working with acetonitrile containing large amounts of water leads to the formation of hydrogen, working with acetonitrile containing only a small amount of water results in the formation of oxalate. Additionally, there is an optimum electrolyte composition where formate is produced with Faradaic efficiencies of approximately 70 %. Díaz‐Duque et al. have further explored acetonitrile‐water mixtures, by employing these mixtures as electrolyte for the electrochemical reduction of CO_2_ on nano‐structured and flat copper electrodes.^94^ They observe optimal CO_2_ reduction currents with a water molar fraction of 0.25.

For ionic liquids, the addition of different amounts of water is quite common and is discussed as well in section 3.1. Rosen et al.^92^ investigated the limitations of water addition to ionic liquids and show that additions of up to 30 mol/liter of water to EMIm‐BF_4_ do not significantly influence amount of hydrogen produced. This demonstrates the inhibiting effect of EMIm‐BF_4_ on the hydrogen evolution reaction, and the favorable effect towards product formation from CO_2_ reduction. The authors hypothesize that [EMIm]^+^ blocks the electrocatalytic surface, preventing hydrogen absorption from the water present in the electrolyte. Therefore, the protons available in the electrolyte are used exclusively for CO_2_RR. However, when more than 30 mol/liter of water is added to the ionic liquid, this saturates the system and the hydrogen evolution reaction is promoted. A similar effect has also been observed in other studies.^90^

Utilizing electrolyte mixtures containing water and ionic liquids can significantly alter the obtained product distribution on metallic electrodes. Huan et al. show that formic acid is the primary product of CO_2_ reduction on a copper dendrite electrode in an electrolyte mixture of [EMIm]BF_4_ and water (92–8 % v/v).^96^ The high Faradaic efficienty of 87 % is remarkable, formic acid is a minor product of CO_2_ reduction on copper electrodes in aqueous electrolytes.^11^

**Figure 6:**
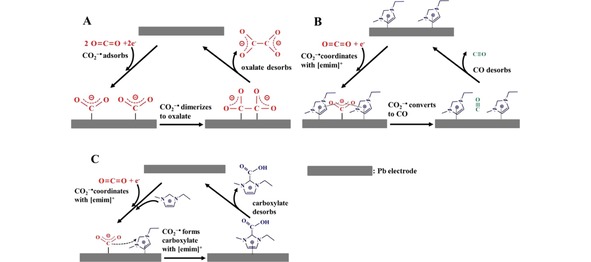
Reaction pathways for the electrochemical reduction of CO_2_ on Pb in an acetonitrile electrolyte, a) without the presence of EMIm‐NTf_2_, and b) and c) in the presence of EMIm‐NTf_2_. Reprinted with permission from Sun et al, *Langmuir* **2014**, *30*, 6302–6308.^[94]^ Copyright 2014 American Chemical Society.

Studies with mixtures of imidazolium‐based ionic liquids and acetonitrile have shown that carboxylation of imidazolium could render it as a proton source for CO_2_ reduction. This affects the reaction stoichiometry and can be a viable alternative for water addition to the electrolyte.^64^ Sun et al. studied the influence of imidazolium‐based ionic liquids on CO_2_ reduction in acetonitrile using Pb electrodes. NMR tests showed that the addition of imidazolium cations stabilizes CO_2_^.−^ intermediates, ultimately leading to CO production (see Figure 6). This stabilization prevents CO_2_^.−^ dimerization to oxalate that would occur without [EMIm]^+^ cations present in the acetonitrile electrolyte.^98^ Additionally, CO_2_^.−^ intermediates can react with the [EMIm]^+^ cation to form a carboxylate adduct.

Ternary electrolyte composition have been reported as well. Studies developed by Zhu et al.,^99^ found that CO_2_ reduction in mixtures of ionic liquids, organic solvent and water using Sn and Pb electrodes led to the conversion of CO_2_ to HCOOH with a Faradaic efficiency of 91.6 %, higher than reported for the same reaction carried out using a non‐mixed electrolyte.^79^ Here, the addition of small amounts of H_2_O, as proton donor, enhanced CO_2_ reduction reaction rates. Wu et al. explored the electrochemical reduction of CO_2_ to formic acid on different metal oxide electrodes in mixtures of imidazolium‐based ionic liquids, water and acetonitrile.^100^ Optimal formic acid production, with a Faradaic efficiency of 95.5 % at an applied potential of −2.3 V vs. Ag/Ag^+^, was achieved by employing a PbO_2_ electrode in an electrolyte consisting of 14.6 wt % [Bzmim]BF_4_ and 11.7 wt % H_2_O in acetonitrile.

## 5 Summary and Outlook

In this minireview, we have discussed how the electrolyte plays an important role in determining the outcome of the electrochemical reduction of CO_2_. In aqueous electrolytes, a pH gradient between the local pH at the surface and the bulk pH of the electrolyte affects the electrochemical reduction of CO_2_ and the hydrogen evolution reaction allowing for the control of product distribution and product selectivity by tuning the local pH at the surface. This principle has been exploited in alkaline electrolysers for CO_2_ reduction that produce C_2_ products, such as ethylene and ethanol, with high Faradaic efficiencies at high current densities and low overpotentials. The addition of specific anions and cations provides another way to tune the selectivity of electrochemical CO_2_ reduction. Although the exact mechanism by which cations and anions alter the CO_2_ reduction reaction is still debated, there is a significant effect on both product selectivity and electrode stability.

Non‐aqueous electrolytes generally have a higher CO_2_ solubility, making them attractive alternatives to aqueous systems. Although generally stable, it is important to test the electrolyte stability and to measure if the carbon atoms in the measured products are coming from CO_2_ or from the electrolyte. The addition of water to non‐aqueous electrolytes, or electrolyte mixing, has a significant effect on the observed product distribution and product stability.

Overall, the electrolyte can be used as a design parameter to enable the selective reduction of CO_2_ to desired products. By controlling the processes taking place at the interface of the electrocatalyst and the electrolyte, better electrocatalytic system can be obtained. Although most work has been performed in aqueous electrolytes, non‐aqueous electrolytes provide additional possibilities to supress the unwanted hydrogen evolution reaction and to enhance CO_2_ reduction due to their higher CO_2_ solubility. By mixing, electrolytes can be engineered to tune the obtained product distribution and product selectivity.

## Conflict of interest

The authors declare no conflict of interest.

## Biographical Information

*Marilia Moura de Salles Pupo received her PhD from Universidade Tiradentes and is currently a postdoctoral researcher at TU Delft in the Large‐Scale Energy Storage section of the Process & Energy department. Her main research focus is on developing new electrolyte compositions for the reduction of CO_2_*.

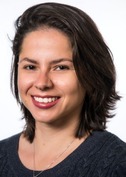

## Biographical Information

*Ruud Kortlever received his PhD from Leiden University and, after a postdoctoral stay at Caltech, is currently an assistant professor at TU Delft in the Large‐Scale Energy Storage section of the Process & Energy department. His group is interested in electrochemical conversions relevant for renewable fuel production and the electrification of the chemical industry*.